# Hyperhomocysteinemia in a Patient With Subdural Hematoma and Subarachnoid Hemorrhage Secondary to a Traumatic Brain Injury

**DOI:** 10.7759/cureus.63792

**Published:** 2024-07-04

**Authors:** Hafiza A Elias, Khadija Mohib, Muhammad Asjad Saleem, Laraib Shabbir Rajput, Aayat Ellahi

**Affiliations:** 1 Department of Medicine, Teklehaimanot General Hospital, Addis Ababa, ETH; 2 Department of Family Medicine, Tarzana Medical Group, Los Angeles, USA; 3 Department of Nephrology, Indus Hospital & Health Network, Karachi, PAK; 4 Department of Medicine, Dow University Hospital, Karachi, PAK; 5 Department of Community Medicine, Jinnah Sindh Medical University, Karachi, PAK

**Keywords:** subdural hematoma, subarachnoid hemorrhage, hyperhomocysteinemia, homocysteine, traumatic brain injury

## Abstract

Traumatic brain injury (TBI) is a significant global health issue, contributing substantially to mortality and disability. Serum biomarkers, such as homocysteine (Hcy), play a critical role in the prognosis of brain injuries, with hyperhomocysteinemia (HHcy) potentially leading to neurological disorders. We present the case of a 64-year-old patient admitted to the emergency department following a road traffic accident (RTA). Magnetic resonance imaging (MRI) revealed parietal subdural hematoma (SDH), right frontal contusion, and left subarachnoid hemorrhage (SAH). The patient underwent a craniotomy to address SAH and SDH. Initial Hcy levels were markedly elevated compared to post-operative levels. Hcy represents a rapid, non-invasive, and cost-effective diagnostic tool for assessing brain injury severity and guiding medical intervention. Early detection of HHcy could potentially mitigate vascular and neurological complications, thereby improving patient outcomes.

## Introduction

Traumatic brain injury (TBI) remains a leading cause of death and disability globally, posing a significant public health challenge [1]. Annually, it is estimated that approximately 69 million individuals sustain TBIs worldwide [2]. Contrary to previous assertions, pathological events such as hypoxia, inflammation, and oxidative stress, which are associated with neuronal injury, can indeed be inferred from imaging studies and are instrumental not only for therapeutic management but also for prognostic evaluation [3].

Serum biomarkers, including homocysteine (Hcy), are critical in assessing the prognosis of brain injuries. Elevated Hcy levels, through their role in the catabolism of choline and folate, are crucial for maintaining methionine activity and protein synthesis. These elevated levels contribute to the production of reactive oxygen species, resulting in cellular damage through mechanisms such as lipid peroxidation [4,5].

Hyperhomocysteinemia (HHcy), often identified through elevated Hcy levels (15 micromol/L) in blood tests [6], is linked to significant neurological, cardiovascular, and immunological disorders. Factors contributing to elevated Hcy include smoking, a diet rich in methionine, and a sedentary lifestyle [6]. Importantly, Hcy exacerbates the permeability of the blood-brain barrier, compromises vascular integrity, and accelerates atherosclerosis [7]. The disruption in trans-sulphuration and re-methylation processes particularly affects the hippocampus's pyramidal neurons, making them susceptible to damage [6]. Furthermore, elevated Hcy levels have been associated with the aggravation of various neurological conditions including epilepsy, Parkinson's disease, and brain atrophy [7].

In this paper, we present a case study of a patient who exhibited elevated Hcy levels following a TBI. Additionally, we review literature assessing neurological outcomes based on serum Hcy levels in patients who have experienced head trauma leading to hemorrhages, strokes, or infarctions.

## Case presentation

A 64-year-old male was admitted to the emergency department after a road traffic collision involving a motorcycle, which occurred nine hours prior to admission on September 2, 2022. The individual, who was a pedestrian at the time, was struck by the motorcycle after the rider lost control. After the accident, the patient returned home but soon experienced disorientation and vomiting, prompting his admission to the hospital due to altered consciousness and repeated vomiting.

Upon examination, the patient's lungs were clear. The patient's pupils were symmetrical and measured 3 mm in diameter, responding equally to light. His level of consciousness was evaluated using the Glasgow Coma Scale, recording a score of 8/15 at admission. He was promptly admitted to the ICU of the Neurosurgery Department for further management. Initial orders included a complete blood count, serum electrolytes, urea, and coagulation profiles. Diagnostic imaging was initiated with a head CT to determine the extent of the injury. A Foley catheter and nasogastric tube were inserted by a house officer. The patient's family was advised to secure two units of whole blood. A follow-up magnetic resonance imaging (MRI) scan conducted four hours later confirmed a parietal subdural hematoma (SDH), a right frontal contusion, and a left subarachnoid hemorrhage (SAH).

Initial laboratory tests including measurements for urea, electrolytes, liver function, prothrombin time, and clotting profiles were within normal limits at the time of admission. However, by September 7, 2022, a notable deviation in the patient's blood parameters was observed. Specifically, hemoglobin levels were slightly below the normal range at 13 mg/dl (normal 14-17 mg/dl), and hematocrit was recorded at 37.4%, falling below the typical range of 42-58%. Additionally, the patient exhibited thrombocytopenia and an elevated erythrocyte sedimentation rate (ESR), indicating an inflammatory response or potential secondary complications.

The patient underwent a craniotomy on September 9, 2022, to address the SAH and SDH. Serum Hcy levels were closely monitored, with initial assessments at admission and subsequent evaluations before discharge. After obtaining consent from the patient's next of kin, a nurse drew 5 milliliters of blood from the accessible median cubital vein, ensuring the sample was correctly labeled and securely transported for analysis. The findings indicated elevated Hcy levels upon admission, which notably decreased post-operatively, suggesting a response to the surgical intervention and ongoing management. These measurements were essential in understanding the biochemical impacts of TBI and the efficacy of treatment approaches (Table 1).

**Table 1:** Serum Hcy levels at admission, pre-operative, and post-operative Hcy: homocysteine

Timeline	Hcy levels (μmol/L)
On admission	27.5
Pre-operative	31.6
Post-operative	16.2

Post-surgery, his condition improved markedly over the following two days. A follow-up MRI confirmed the successful mitigation of the initial brain injuries. His Glasgow Coma Scale score improved from 8/15 upon admission to 13/15 after the procedure, reflecting a significant recovery in his level of consciousness. He was subsequently discharged on September 11, 2022, with improved neurological status. The patient did not return for scheduled follow-up visits. 

## Discussion

The current study's findings resonate strongly with prior research that demonstrates elevated plasma Hcy levels in patients suffering from TBI compared to control groups. This is aligned with Rahmani et al., who observed significantly higher Hcy levels in the brain trauma group compared to controls [5]. Furthermore, this study establishes a noteworthy correlation between plasma Hcy levels and both the Marshall score and the Glasgow Outcome Scale (GOS), with statistically significant p-values of 0.028 and 0.001, respectively. These scores are instrumental in evaluating the severity of brain injuries (Marshall score) and the subsequent recovery outcomes (GOS), suggesting that Hcy levels could serve as a predictive biomarker for both initial trauma severity and long-term neurological recovery [5].

Our analysis further delves into the impact of Hcy on cognitive functions. According to assessments such as the Mini-Mental State Examination and measures of cerebral hemodynamic status via pulsatile indices, we found robust evidence of a linkage between elevated Hcy levels and cognitive dysfunction (p=0.0001) [8]. This aspect of the research highlights the multifaceted impacts of metabolic disturbances on brain function following injury, pointing towards secondary cognitive impairments that may complicate recovery processes.

Additionally, our study touches upon the broader implications of systemic responses post-TBI. Elevated levels of C-reactive protein (CRP) have been associated with poorer outcomes following ischemic attacks, significantly heightening the risk of subsequent strokes [9]. Moreover, increased levels of vascular endothelial growth factor, crucial in angiogenesis, have been documented in patients with post-acute ischemic stroke [10]. These findings suggest that systemic inflammatory and angiogenic responses play critical roles in the pathology and recovery of neurological functions post-TBI.

An essential component of managing TBI involves the initial assessment of the injury's severity. Traditional techniques such as CT scans and cognitive tests like the Glasgow Coma Scale are foundational. However, our findings, along with supporting literature, suggest these methods might not sufficiently predict long-term prognoses and neurological outcomes on their own [11]. This highlights the necessity for a multimodal approach that incorporates biochemical markers like Hcy alongside radiological and cognitive assessments to refine prognostic accuracy and therapeutic strategies.

In light of these observations, we recommend integrating biochemical profiling, particularly plasma Hcy levels, in routine TBI assessments to better predict outcomes and tailor therapeutic interventions. Given the complex interplay of factors influencing recovery in TBI patients, ranging from genetic predispositions to lifestyle factors and immediate medical interventions, understanding and integrating these biomarkers into clinical protocols could significantly enhance patient management and outcome predictions.

## Conclusions

This study has highlighted the significant association between elevated plasma Hcy levels and the severity of TBIs. Considering the role that Hcy plays in the pathogenesis of TBI, routinely measuring Hcy levels in TBI patients during clinical assessments may improve prognostic accuracy and allow for more individualized treatment approaches. This work also supports a multimodal approach to TBI care, combining conventional imaging methods with biochemical markers to offer a thorough assessment of injury severity and recovery potential. 
